# Left ventricular assist device and pump thrombosis: the importance of the inflow cannula position

**DOI:** 10.1007/s10554-022-02683-z

**Published:** 2022-07-19

**Authors:** Kirsten A. Kortekaas, Michiel A. de Graaf, Meindert Palmen, Jerry Braun, Bart J. A. Mertens, Laurens F. Tops, Saskia L. M. A. Beeres

**Affiliations:** 1 Department of Cardiology, Leiden University Medical Center, Leiden, The Netherlands; 2 Department of Cardiothoracic Surgery, Leiden University Medical Center, Leiden, The Netherlands; 3 Department of Statistics, Leiden University Medical Center, Leiden, The Netherlands

**Keywords:** CT based angle measurement, Left ventricular assist device, Pump thrombosis

## Abstract

Pump thrombosis is a devastating complication after left ventricular assist device implantation. This study aims to elucidate the relation between left ventricular assist device implantation angle and risk of pump thrombosis. Between November 2010 and March 2020, 53 left ventricular assist device-patients underwent a computed tomography scan. Using a 3-dimensional multiplanar reformation the left ventricular axis was reconstructed to measure the implantation angle of the inflow cannula. All patients were retrospectively analyzed for the occurrence of pump thrombosis. In 10 (91%) patients with a pump thrombosis, the implantation angle was towards the lateral wall of the left ventricle. In only 20 patients (49%) of the patients without a pump thrombosis the inflow cannula pointed towards the lateral wall of the left ventricle. The mean angle in patients with a pump thrombosis was 10.1 ± 11.9 degrees towards the lateral wall of the left ventricle compared to 4.1 ± 19.9 degrees towards the septum in non-pump thrombosis patients (P = 0.005). There was a trend towards a significant difference in time to first pump thrombosis between patients with a lateral or septal deviated left ventricular assist device (hazard ratio of 0.15, P = 0.07). This study demonstrates that left ventricular assist device implantation angle is associated with pump thrombosis. Almost all patients in whom a pump thrombosis occurred during follow-up had a left ventricular assist device implanted with the inflow-cannula pointing towards the lateral wall of the left ventricle.

## Introduction

Developments in the field of mechanical circulatory support for end-stage heart failure have resulted in an increased use of left ventricular assist devices (LVAD), especially as *destination* therapy [1]. The excitement derived from improved survival after LVAD implantation, however, has been tempered by the risk of devastating complications including major bleedings and pump thrombosis (PT). Accordingly, reducing thrombogenic potential and thereby improving long-term outcomes is a major research topic.

Recently, the relation between PT and the angulation of the left ventricular (LV) inflow cannula has gained interest since the angulation is thought to affect intraventricular flow dynamics, in specific flow obstruction, which can predispose thrombus formation. Various hemodynamic simulations have been published to model the flow through the LV into the inflow cannula suggesting a relation between axis deviation and increased thrombogenicity [2–5].

Clinical investigations of inflow cannula position are scarce. Two previous studies showed that the cannula in patients with low incidence of PT was aligned parallel to the intraventricular septum [6] and directed at the mitral valve [7]. Most probably, this alignment promotes better flow patterns and efficient LV unloading. In a third study, Sorensen et al. showed that malpositioning towards the intraventricular septum might contribute to PT [8]. However, in this study only 2 of the 68 (2.9%) patients developed PT.

The present study aims to explore the relation between inflow cannula angulation and PT in the clinical setting. We hypothesize that deviations of the LVAD inflow cannula alignment lead to an increased risk of PT by means of unfavorable hemodynamics.

## Materials and methods

### Patient population

All 79 patients in whom a continuous flow LVAD (HeartWare Inc., Framingham, MA) was implanted as *destination* therapy for heart failure in the Leiden University Medical Center, between November 2010 and March 2020 were eligible for inclusion. Our center has a registration for *destination* therapy only. For the current study, patients in whom no computed tomography (CT) scan was performed after LVAD implantation were excluded (n = 26). Of these 26 patients only 1 patient had a PT. For the present analysis patients with a congenital heart disease requiring a ventricular assist device were not included. Informed consent was obtained from all included patients that were alive when the study was conducted. The study protocol was reviewed by the local medical ethics committee (G20.182) who waived the need for official approval according to the Medical Research Involving Human Subjects Act.

### Data analysis

Clinical, laboratory and survival data were collected and analyzed retrospectively from the patient information systems (EPD-Vision; Leiden University Medical Center, Leiden, the Netherlands; HiX 6.1, Chipsoft, Amsterdam, the Netherlands). Baseline variables, including etiology of heart failure and Standard Interagency Registry for Mechanically Assisted Circulatory Support (INTERMACS) classification, were collected at date of LVAD implantation. Medical history was screened for the occurrence of PT. PT was defined as ≥ 2 signs or symptoms of PT in combination with an accompanying intervention such as intensified treatment with anti-coagulation (standard regimen consisted of vitamin K antagonist in combination with clopidogrel), intravenous thrombolytics or pump replacement. The following signs and symptoms were considered suggestive of PT in line with the consensus document by Kormos et al.: (1) presence of hemolysis, (2) worsening of heart failure and (3) abnormal pump parameters [9, 10]. Any available CT visualizing the thorax that was performed for any clinical indication during follow up, was used for measurement of LVAD implantation angle.

### CT measurements

**Fig. 1:**
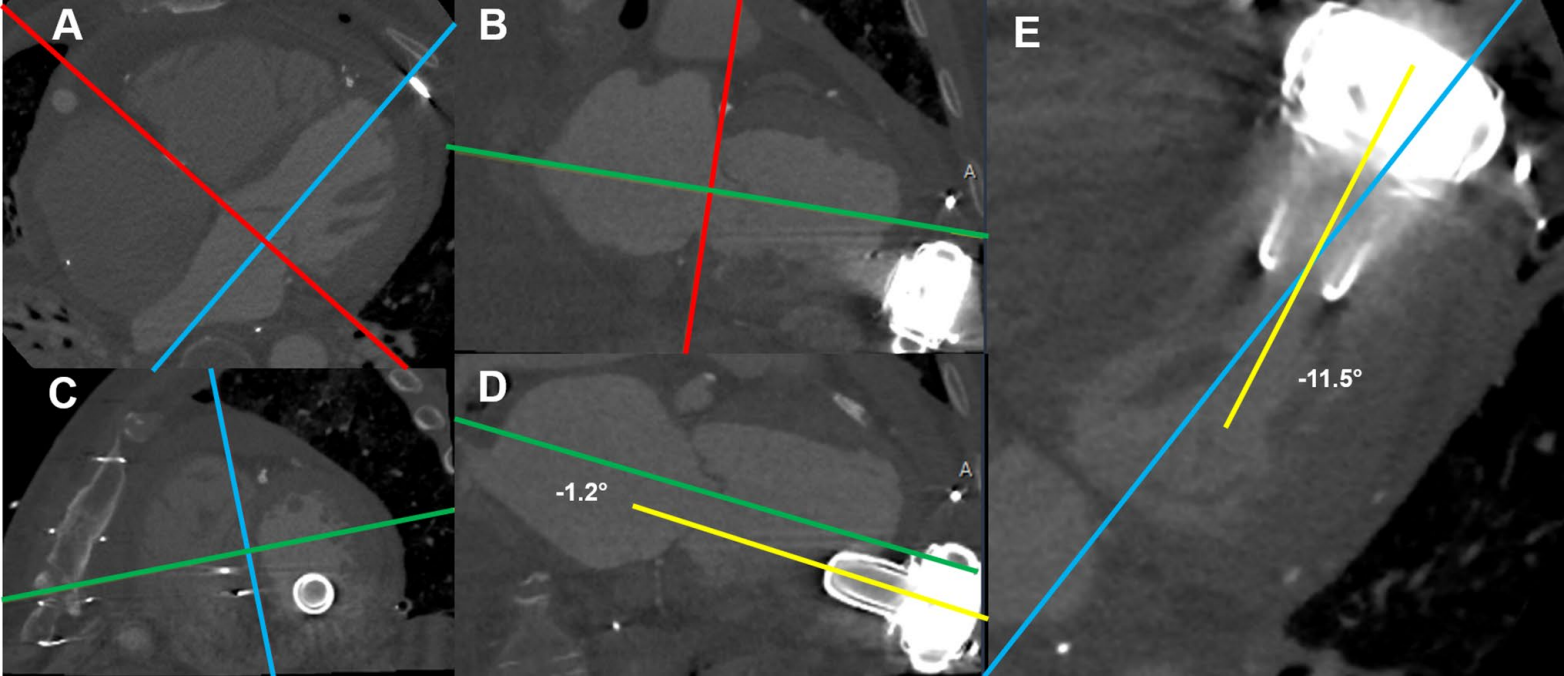
Example of Computed Tomography measurements. Computed tomography data were used to measure the implantation angle of the inflow cannula of the LVAD. Using a 3-dimensional MPR the LV long axis was reconstructed. **A** On a transversal plane, the MPR crosshair is aligned with the mitral valve annulus and the perpendicular part of the crosshair parallel to the LV long-axis. **B** This is repeated in the sagittal view to create a double oblique short-axis view of the LV. **C** The crosshair is further aligned to the septum to create a double oblique four-chamber and two-chamber view, with the crosshair parallel to the LV long-axis. (D + E) By adjusting the window settings the angle between the inflow cannula of the LVAD and the LV-axis can be visualized in the two-chamber (**D**; anterior or inferior) and four-chamber (**E**; septal or lateral) view. *LV* left ventricle, *LVAD* left ventricular assist device, *MPR* multiplanar reformation, *PT* pump thrombosis

Non-ECG gated CT data sets were used to measure the implantation angle of the inflow cannula of the LVAD. The LV long axis was reconstructed using a 3-dimensional multiplanar reformation (MPR) (Fig. 1). First, on a transversal plane the MPR crosshair was aligned with the mitral valve annulus and the perpendicular part of the crosshair parallel to the LV long-axis (Panel A). This was repeated in the sagittal view to create a double oblique short-axis view of the LV (Panel B). Then, the crosshair was further aligned to the septum (Panel C) to create a double oblique four-chamber and two-chamber view, with the crosshair parallel to the LV long-axis. By adjusting the window settings (width 3000 Hounsfield units, level 800 Hounsfield units) the inflow cannula of the LVAD was clearly visualized (Panel D and E). The angle between the inflow cannula and the LV-axis was then measured in the four-chamber (Panel E) (septal or lateral) and two-chamber view (Panel D) (anterior or inferior). To improve reproducibility the septum was mainly used as a reference instead of the neo-apex which might be influenced as a result of the surgical procedure. Measurements were performed by two experienced observers (MG and KK), in two different readings. The results were compared to assess the inter-observer variability. For the final analysis the average between the two measurements was used. After a period of several months one observer (MG) re-measured all cases to assess intra-observer variability blinded to initial results.

### Follow-Up/Statistical analysis

Continuous variables are expressed as mean ± SD when normally distributed or otherwise as the median and interquartile range (IQR). Categoric variables are presented as numbers and percentages. The implantation angle of patients with and without PT was compared. Comparison of continuous data was performed using two-tailed unpaired Student *t* test for normally distributed variables.

Patients were included in the current study on the date of LVAD implantation. The first CT scan visualizing the thorax after LVAD implantation was used for the measurements of the implantation angle. All included patients had a CT scan before occurrence of a PT. For the present study we assumed that the angle of the LVAD did not change over time. Therefore, the measurement on the date of CT was used as a proxy for the angle on the date of implant. Time to first PT from study entry at date of LVAD implantation was compared between patients with a septal versus a lateral deviated LVAD. To account for competing risks, we carried out a cause specific hazard analysis for the time from implant to first PT based on a Cox regression analysis. The extent of agreement between the two measurements of the implantation angle of the inflow cannula of the LVAD was assessed with the Bland–Altman test and intraclass correlation coefficient for both inter and intra-observer variability. *P* values less than 0.05 were considered statistically significant and are marked bold in the tables. Statistical analysis was performed using SPSS 25.0 software (IBM, Armonk, NY).

## Results

### Patient population

The baseline characteristics of the 53 included patients are depicted in Table 1 and 2. Mean age was 62.4  ±  9.2 years and 40 patients (75%) were male. The majority of patients had an ischemic etiology of their heart failure. There were no significant differences in baseline characteristics between patients with and without pump thrombosis except that patients with pump thrombosis had a higher Log EuroScore and more frequently diabetes mellitus. In most patients (94%), a median sternotomy was used for LVAD implantation. The median time between the date of LVAD implantation and the scan used for assessment of the LVAD inflow cannula position was 83 days (IQR 15–656). Of the 53 CT scans used, 32 were contrast-enhanced (60%).

**Table 1:** Baseline characteristics

Baseline characteristics	Total (N = 53)	PT (N = 11)	No PT (N = 42)	P-value
Age (years)	62.4 ± 9.2	62.3 ± 8.5	62.5 ± 9.5	0.955
Gender (male)	40 (75%)			
BMI (kg/m^2^)	26.0 ± 3.6	27.0 ± 2.9	25.6 ± 3.8	0.334
Log EuroScore (%)	20.0 (IQR 10.0–33.5)	29.0 (IQR 23.0–47.0)	18.5 (IQR 9.75–28.0)	**0.035**
INTERMACS profile				NA
1.Critical cardiogenic shock	2 (4%)	0	2 (5%)	
2.Progressive decline	3 (6%)	1 (9%)	2 (5%)	
3.Stable but inotrope dependent	27 (51%)	6 (55%)	21 (50%)	
4.Resting symptoms	9 (17%)	1 (9%)	8 (19%)	
5.Exertion intolerant	11 (21%)	3 (27%)	8 (19%)	
6.Exertion limited	0	0	0	
7.Advanced NYHA class 3	1 (2%)	0	1 (2%)	
Etiology of heart failure				0.804
Ischemic cardiomyopathy	32 (60%)	7 (64%)	25 (60%)	
Non-ischemic cardiomyopathy	21 (40%)	4 (36%	17 (41%)	
Left ventricular ejection fraction before implant (%)	23 (18–29)	27 (23–30)	21 (17–29)	0.062
Left ventricular end-diastolic diameter (mm)	67 (64–73)	70 (67–78)	67 (63–73)	0.079
Previous thoracotomy	25 (47%)	7 (64%)	18 (43%)	0.219
Previous atrial fibrillation	23 (43%)	6 (55%)	17 (41%)	0.402
Previous ischemic stroke	4 (8%)	0 (0%)	4 (10%)	0.287
Previous venous thromboembolism	4 (8%)	0 (0%)	4 (10%)	0.287
Previous diabetes mellitus	11 (21%)	5 (46%)	6 (14%)	**0.023**
Cardiac resynchronization therapy	38 (72%)	9 (82%)	29 (69%)	0.403

*INTERMACS* Interagency Registry for Mechanically Assisted Circulatory Support, *NYHA* New York Heart Association, *PT* pump thrombosis

Patient data at baseline. Values are shown as mean ± SD, n (%) or median (interquartile range)

**Table 2:** Surgical characteristics

Surgical characteristics	Total (N = 53)	PT (N = 11)	No PT (N = 42)	P-value
Median sternotomy	50 (94)	9 (82)	41 (98)	**0.044**
Concomitant surgery	47 (89)	9 (82)	38 (90)	0.420
Tricuspid valve annuloplasty	38 (72)	7 (64)	31 (74)	0.505
LAA excision	31 (59)	4 (36)	27 (64)	0.094
Left ventricular reconstruction	9 (17)	2 (19)	7 (17)	0.905
Aortic valve replacement	8 (15)	2 (19)	6 (14)	0.748
PFO/ASD closure	2 (4)	0 (0)	2 (5_	0.461

*ASD* Atrial Septal Defect, *LAA* left atrial appendage, *PFO* patent foramen ovale, *PT* pump thrombosis.

Surgical data at baseline. Values are shown as n (%)

### Clinical follow-up

During a median follow-up of 582 days (IQR 208–1561), 38 patients (72%) died with a median time from implant to death of 478 days (IQR 59–1378). In total, eleven of the 53 patients experienced one or more LVAD PT. Median time from LVAD implantation to first PT was 332 days (IQR 74–651). INR values of patients who experienced a PT were all above 2.0 (2.2–4.3) except for two patients. One patient used therapeutic low molecular weight heparin because of recent hemorrhagic stroke (oral anticoagulation was discontinued). Another patient used unfractionated heparin because he was still admitted to the intensive care unit after LVAD implantation (APTT 63–72).

### LVAD implantation angle measurement: inter and intra-observer variability

**Fig. 2:**
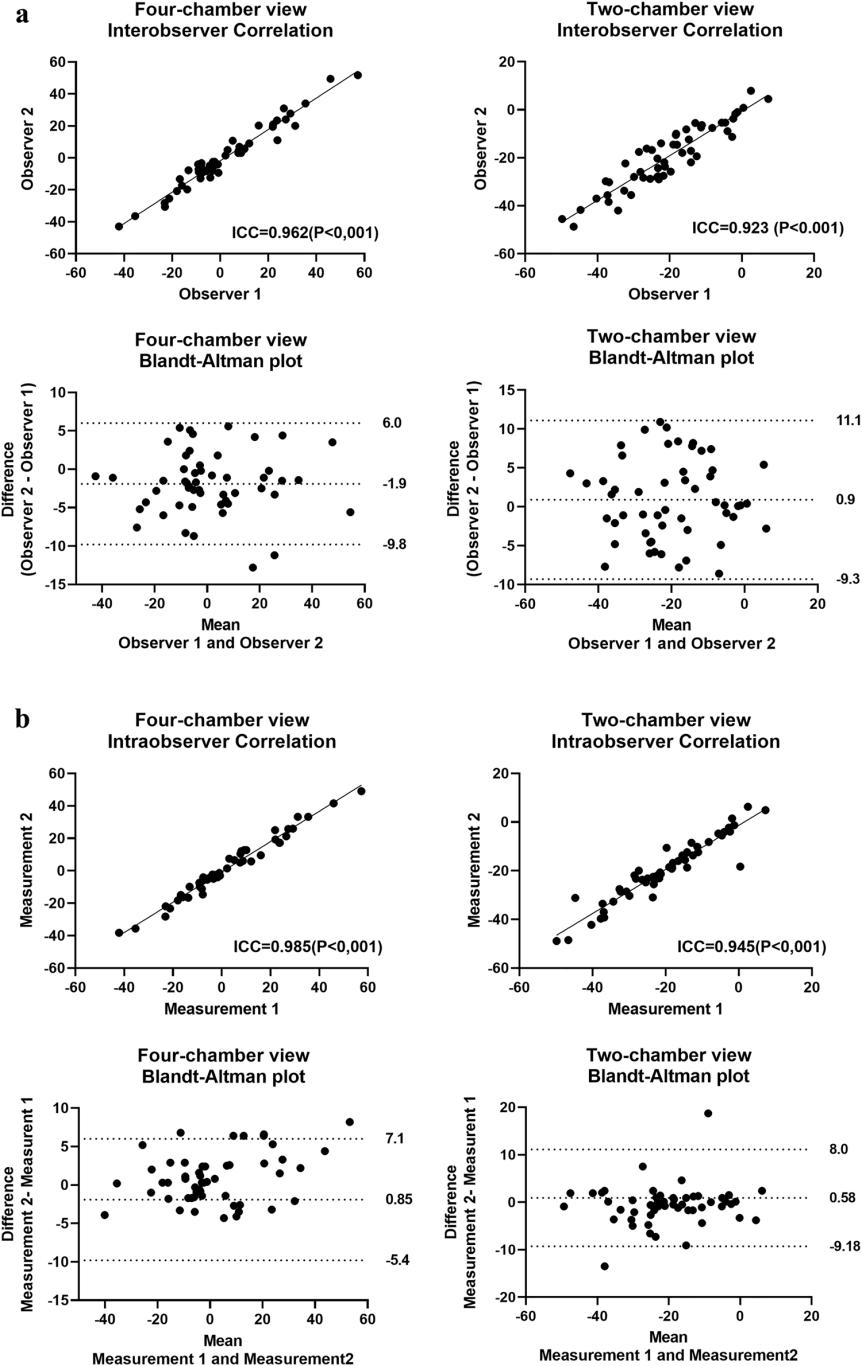
Bland–Altman analysis and correlation plots. **a** Bland–Altman and correlation plots showing inter-observer agreement for CT measurements in all subjects (n = 53). On the four-chamber view a negative value represents an angle towards the lateral wall of the LV. On the two-chamber view a negative value represents an angle towards inferior. **b** Bland–Altman and correlation plots showing intra-observer agreement for CT measurements in all subjects (n = 53)

The CT scans of all 53 patients were reviewed by two independent observers (Fig. 2a). In one patient the angle could not be assessed on the four-chamber view by both reviewers due to insufficient scan range; this patient did not have a PT. Correlation for the implantation angle between the observers on the four-chamber view was 0.962 (P < 0.001) and on the two-chamber view 0.923 (P < 0.001). Bland–Altman analysis for the four-chamber view demonstrated a bias of − 1,9 degrees with limits of agreement ranging from − 9.8–6.0 degrees. Similarly, for the two-chamber view the bias was 0.9 degrees with limits of agreement ranging from − 9.2–11.1 degrees. Similar results were observed for intra-observer variability. (Fig. 2b). No clear differences were observed in reproducibility between contrast or non-contrast enhanced CT-scans (data not shown).

### LVAD implantation angle

The LVAD implantation angle was assessed on the four-chamber view. Of the 52 patients, 22 (42%) had an LVAD implanted towards the LV septum and 30 (58%) towards the LV lateral free wall. Overall, the mean implantation angle on the four-chamber view was 1.0  ± 1.9 degrees towards the septal wall. On the two-chamber view in 50 patients (94%) the LVAD implantation angle on the two-chamber view was towards inferior, whereas in only 3 (6%) patients the LVAD was facing the anterior wall of the LV. Overall, on the two-chamber view the angle was 20.1 ± 12.8 degrees towards the inferior wall.

### Relation between PT and implantation angle

**Fig. 3:**
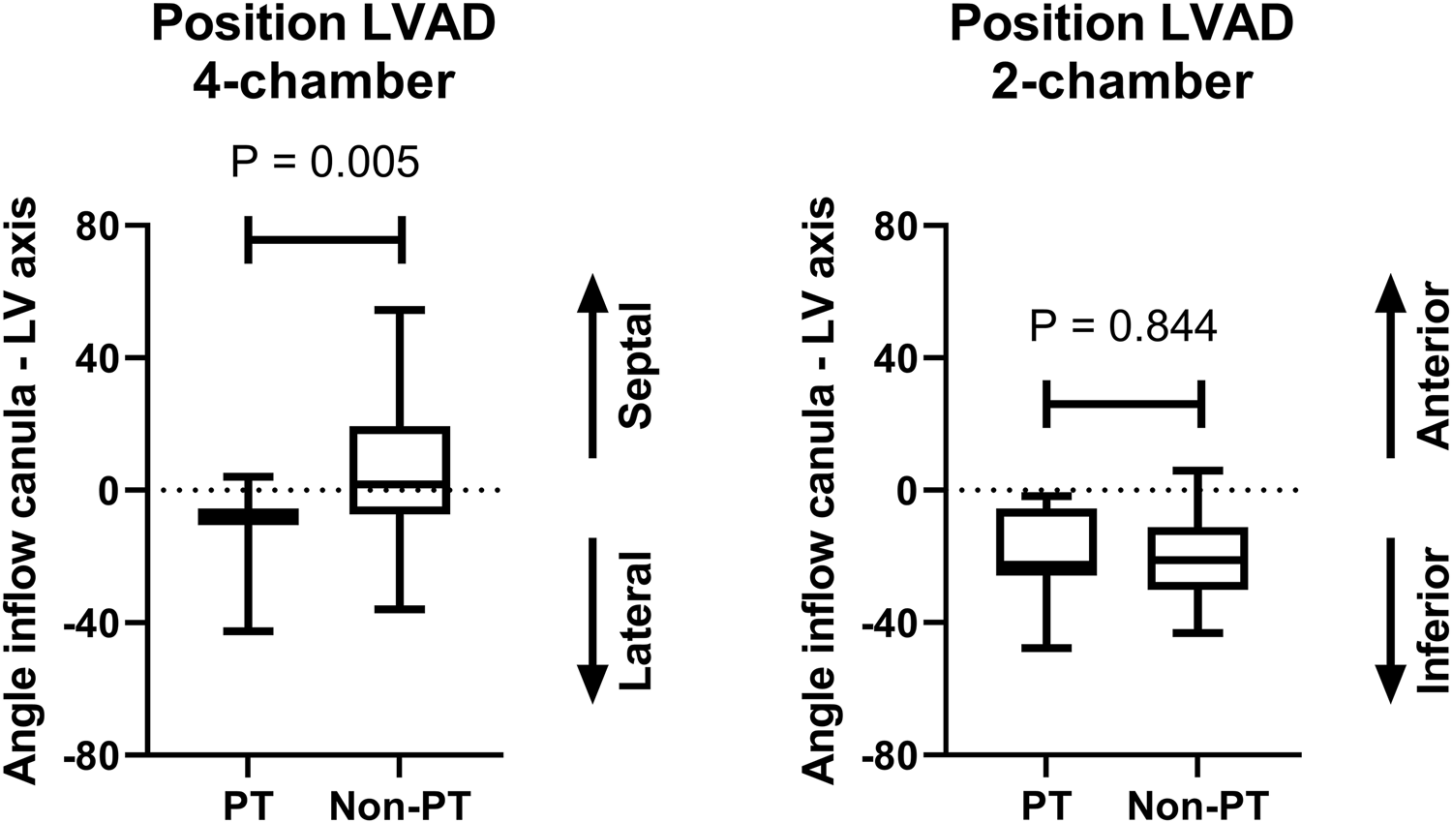
Mean implantation angle. Boxplot graph demonstrating the relation between PT and implantation angle. Overall, 42% of the patients had an LVAD implanted towards the LV septum and 58% towards the lateral wall of the LV. In patients with a pump thrombosis 91% had an implantation angle towards the lateral wall of the LV compared to 49% of the non-pump thrombosis (non-PT) patients. On the two-chamber view the LVAD implantation angle was towards inferior in the majority of patients (94%). None of the patients with a pump thrombosis had an implantation angle towards anterior. *LVAD* left ventricular assist device, *PT* pump thrombosis

**Fig. 4:**
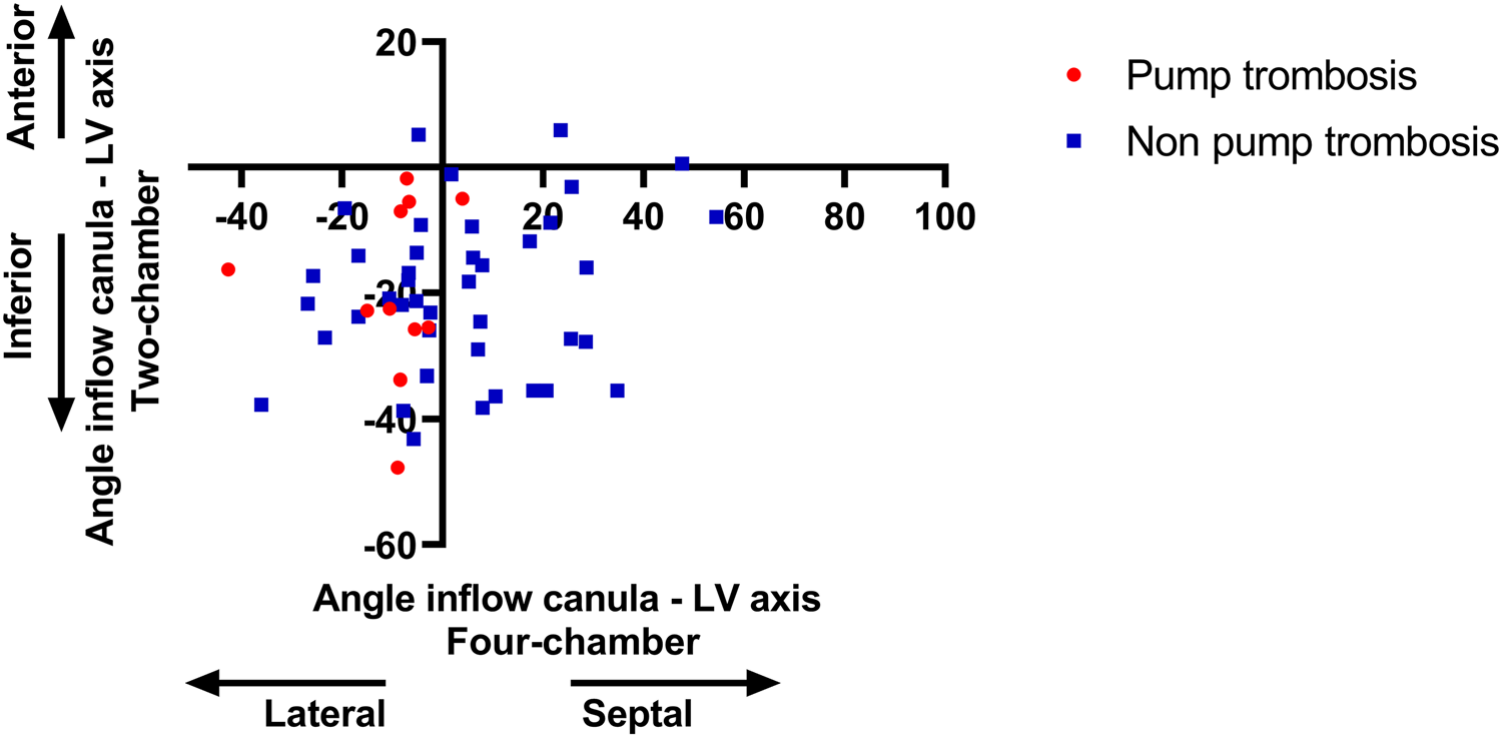
Relation between two- and four-chamber view implantation angle. This scatter diagram shows the relation between the implantation angle on the four- (X axis) and two- (Y axis) chamber view in relation to the occurrence of left ventricular assist device pump thrombosis. Of note, the majority of patients with a pump thrombosis had an implantation angle towards the inferolateral wall of the LV. *LV* left ventricle

To assess the association between implantation angle and PT, a comparison was made between the 11 patients with and the 42 patients without PT. On the four-chamber view, in the 11 patients with PT, 10 patients (91%) had an implantation angle towards the lateral wall of the left ventricle (LV). Interestingly, of the patients without PT, only 20 patients (49%) had an implantation angle towards the lateral wall of the LV (P = 0.012). As depicted in Fig. 3, the mean angle in patients with an LVAD PT was 10.1 ± 11.9 towards the lateral wall of the LV. In the non-PT group the mean angle was 4.1 ± 19.9 towards the septum (P = 0.005). On the two-chamber view, none of the patients with a PT had an LVAD angulated towards the anterior wall. In non-PT patients, only 3 of the 42 patients had an LVAD facing the anterior wall (7%). The mean angle was 19.4 ± 14.1 in patients with a PT compared to 20.3 ± 12.6 for non-PT patients (P = 0.844) (Fig. 3). Figure 4 demonstrates the relation between the implantation angle on the four- and two-chamber view in relation to the occurrence of LVAD PT. Of note, the majority of patients with PT had an implantation angle towards the inferolateral wall of the LV.

Using Cox regression survival analyses, the difference in time between implant to first PT between patients with a lateral or septal deviated LVAD was assessed. With a hazard ratio of 0.15 (95% confidence interval 0.02–1.16) (P = 0.068), there was a trend towards a difference. Septal deviation is associated with a lower hazard ratio.

## Discussion

The main finding of the current study is that LVAD implantation angle, evaluated by 3-dimensional assessment on computed tomography, is associated with PT. Almost all patients in whom a PT occurred during follow-up had an LVAD implanted with the inflow-cannula pointing towards the lateral wall of the LV.

LVAD PT is one of the most feared complications occurring in 2–6% of the patients within the first six months after implant [9, 11]. Treatment remains challenging. Adherence to a structured surgical implant to create an unobstructed blood flow path and clinical management is associated with lower incidence of (early) PT. Survival after LVAD PT is around 70–92% at 30 days [12, 13]. The mortality in our study is higher because our patient population only consisted of *destination* therapy patients and our follow-up period was longer than 30 days.

In the current study, patients with PT had an average angle between the inflow cannula and the LV axis of 10 degrees towards the lateral wall of the LV. This is in line with the findings by Chivukula et al. who used computational fluid dynamics to model the inflow for various inflow cannula angulations in vitro [14]. They concluded that angulation of the inflow cannula > 7 degrees from the apical axis leads to unfavorable hemodynamics and thereby potentially increased thrombogenicity. It should be noted that this was a computational study, not including real patient data. Also, the left ventricular apex position is associated with the occurrence of PT [15]. There is some data on relation between LVAD angle and PT derived from pathology studies suggesting that minor thrombosis of the inflow cannula is frequently observed and unrelated to clinically relevant PT. In a pathologic case serie of eight patients with an LVAD who were successfully bridged to transplant, the position of the cannula was assessed by chest X-rays, CT scans and echocardiography [16]. All patients were found to have thrombus associated with the outer aspect of the LVAD inflow cannula but none had signs of *clinical* PT. Only one patient showed a sub-optimal positioning of the inflow cannula (towards posterior). It is important that these were patients successfully bridged to transplant, so no cases with clinically manifest PT were included. Limited studies on PT and LVAD angle in-vivo are available. In a recent study, 63 patients with an LVAD were followed for one year from index discharge [17]. Cannula coronal angle was measured on chest X-rays and linked to a composite endpoint of stroke and PT. There was no relation with cannula angle and this endpoint. However, only two-dimensional chest X-ray was used. The only study on the relation between PT and dedicated 3-dimensional assessment of LVAD inflow angle was recently published by Sorensen et al. [8] In 68 HeartWare LVAD and 54 HeartMate LVADs they showed that a deviation towards the interventricular septum was associated with an increased risk of thrombosis. However, it should be noted that in their study only 2 PTs occurred in patients with a HeartMate.

Potentially is it not the direction of which the inflow cannula is deviating but merely the deviation itself is related to PT. Noteworthy, during implantation of an LVAD, the surgeon is able to determine the location of the inflow cannula. However influencing the angle between the LV septum and LV inflow cannula in an empty LV during cardiopulmonary bypass is challenging. A solution for this problem might be a gimbaled sewing ring. With this ring, the angle can be adapted for several degrees post-implant [18].

### Strengths and limitations

The main strength of the present analysis is the dedicated 3-dimensional analysis of LVAD inflow cannula implantation angle. Previous studies assessed the LVAD implantation angle on a 2-dimensional anterior posterior chest X-ray [2, 7]. However, the LV is a conical shaped structure, and especially in patients with (dilated) cardiomyopathies, the LV can be extremely deformed with an aberrant position within the thorax. For a dedicated, precise analysis, a 3-dimensional approach is needed. In the present analysis, we assumed that LVAD implantation angle is stable over time. This assumption is supported by two studies by Kazui et al. and Adamson et al., showing a stable position over time using routine chest radiographs in patients with an LVAD [19, 20]. There is no current proof of LVAD position stability on 3-dimensional imaging. Another strength of the present cohort is the fact that it consists of patients in whom an LVAD was implanted as *destination* therapy which allows for long(er) follow-up.

There are some limitations which need consideration when interpreting the results of the present study. First, this is a single-center study and the results need duplication in a larger cohort. Secondly, for this analysis only patients with a HeartWare LVAD were included. Probably the results can be extrapolated to other types of inflow cannulas but this remains to be investigated. The global sale of the HeartWare system has recently been stopped due to observational evidence associated with increased neurological adverse events and mortality. However, ongoing support is necessary for patients who currently have this type of LVAD, approximately 4000 people worldwide. Despite the fact that the present investigation is a retrospective study, there is no loss to follow-up. All patients are followed at the out-patient clinic of the Leiden University Medical Center and informed consent was obtained in all patients. Potentially, bias could have been introduced by the fact that the study only included patients in whom, for clinical purposes, a CT-scan was performed. Only one patient with a PT had no scan in our cohort. Hypothetically in patients with more co-morbidities or worse cardiac functional status more often CT-scans are performed and these co-morbidities might correlate with PT. Moreover, for the present study only static CT scan was available. In none of our patients a full cardiac cycle ECG-gated CTA was available. Therefore, the influence of the cardiac cycle itself on the orientation of the inflow canula in relation to the LV axis can not be evaluated.

## Conclusion

This study, evaluating 3-dimensional LVAD inflow cannula position in the LV by means of computed tomography, demonstrates that angulation deviations are associated with PT. Especially, an inflow cannula position towards the lateral wall of the LV is associated with increased thrombotic risk. This underlines the need for careful positioning of the inflow cannula during LVAD implantation.

## Data Availability

Individual patients data are not available for data sharing. The data that support the findings of this study might be available from the corresponding author upon reasonable request.
